# Youth employment in the primary health care ecosystem in Zambia: barriers and opportunities

**DOI:** 10.3389/frhs.2026.1869977

**Published:** 2026-10-21

**Authors:** Gabriel Mwila, Alice Lakati, Viviane Sakanga, Diana Bulanda, Levy Mkandawire, Jarim Omogi, Lister Banda, Beatrice Gatumia, Osman Abdi, Abdul Agukoh, Micah Matiang’i, Peninah Masibo

**Affiliations:** 1 Amref Health Africa in Zambia, Lusaka, Zambia; 2 Amref International University, Nairobi, Kenya; 3 Amref Institute of Capacity Development, Nairobi, Kenya; 4 Amref Health Africa in Kenya, Nairobi, Kenya

**Keywords:** employment readiness, health systems, health workforce, primary health care, training quality, workforce development, youth employment, Zambia

## Abstract

Youth unemployment remains a critical challenge in Zambia despite increasing demand for health services and investments in primary health care (PHC). While PHC has the potential to generate both health and health-adjacent employment opportunities, limited empirical evidence exists on the institutional and individual factors shaping youth employment readiness within this ecosystem. This study aimed to identify barriers and facilitators influencing youth employment readiness and opportunities in Zambia's PHC sector. A mixed-method, cross-sectional study was conducted in four districts (Lusaka, Kafue, Kitwe, and Ndola). Quantitative data were collected from 859 PHC trainees, interns, volunteers, and early-career professionals using a structured digital questionnaire. Qualitative data were gathered through 64 key informant interviews and 5 focus group discussions, including educators, employers, regulators, and PHC workers. Quantitative data were analyzed using descriptive statistics and multivariate logistic regression with Stata, while qualitative data were thematically analyzed on Atlas.ti. Employment readiness was significantly associated with age, education level, and early-career exposure. Youth aged 25–29 years (OR = 2.05), 30–34 years (OR = 4.31), and ≥35 years (OR = 3.28) were more likely to be employment-ready than those aged 18–24 years. Interns (OR = 2.97) and volunteers/early-career workers (OR = 17.55) had substantially higher odds of readiness compared to students, while respondents with undergraduate education or higher were nearly three times more likely to be employment-ready than diploma holders. No significant sex differences were observed. Qualitative findings identified gaps in training quality, limited clinical exposure, weak regulation, poor working conditions, and constrained career progression, while internships, mentorship, blended learning, and health-adjacent employment pathways emerged as facilitators. Youth employment readiness within Zambia's PHC ecosystem is shaped by education quality, practical exposure, and system-level workforce conditions rather than demographic characteristics alone. Strengthening training regulation, expanding structured school-to-work transition pathways, improving working conditions, and recognizing PHC as a broader employment ecosystem could enhance youth employability while supporting resilient primary health care systems.

## 1 Introduction

Youth unemployment remains one of the most pressing socioeconomic challenges facing sub-Saharan Africa, with profound implications for economic development, social stability, and health system strengthening (1). At the same time, countries continue to experience shortages and inequitable distribution of health workers that undermine progress towards Universal Health Coverage (UHC) and the health-related Sustainable Development Goals (2). Rather than viewing these challenges independently, growing evidence suggests that investments in the health workforce can simultaneously strengthen health systems while creating employment opportunities, particularly within primary health care (PHC), where service delivery depends on a diverse workforce operating across communities and health facilities (3).

Primary health care is particularly important because it relies on multidisciplinary teams that deliver preventive, promotive, curative, rehabilitative, and community-based services across all levels of the health system. In addition to doctors, nurses, and clinical officers, PHC depends on laboratory personnel, pharmacists, nutritionists, environmental health practitioners, community health workers, health educators, programme managers, and other support personnel who contribute to service delivery (2). Beyond direct health occupations, investments in health systems also generate employment in non-health occupations that support service delivery, creating broader economic benefits throughout the health ecosystem (3).

According to the 2022 Census of Population and Housing, Zambia had a population of 19.6 million, of whom approximately 60% were younger than 25 years (4). Despite continued investments in education and skills development, many young people remain unemployed or underemployed, with employment concentrated in urban areas and substantial reliance on the informal economy (5). Although the Government of Zambia has prioritized youth empowerment through the National Youth Policy (6), translating this growing pool of educated young people into productive employment remains a major challenge. At the same time, Zambia continues to face health workforce shortages and projected deficits that threaten the expansion and sustainability of PHC services (7), suggesting that strengthening the PHC workforce may offer opportunities to address both health system and employment priorities.

Employment within the PHC ecosystem encompasses a wide range of occupations with differing educational requirements, career pathways, and employment prospects. While community health workers provide an important entry point into community-based service delivery (8, 9), the PHC workforce also includes skilled and semi-skilled professionals such as nurses, clinical officers, laboratory personnel, pharmacists, nutritionists, environmental health practitioners, and other allied health workers. The present study focuses primarily on trainees, recent graduates, interns, volunteers, and early-career professionals preparing to enter these skilled PHC occupations, while recognizing the complementary contribution of community-based and health-adjacent roles within the broader PHC ecosystem.

Evidence from low- and middle-income countries indicates that workforce development in PHC is influenced by the quality of pre-service education, opportunities for supervised clinical practice, supportive leadership, and effective transition from training into employment (10). Studies have also identified persistent challenges related to workforce motivation, retention, and career progression, particularly in resource-constrained settings (11). In Zambia, research has highlighted challenges in scaling community health worker programmes (9), motivating and retaining PHC workers (12), and strengthening leadership within frontline health services (13). However, relatively little attention has been paid to understanding how these institutional and workforce factors influence employment readiness among young people transitioning from training into the PHC workforce.

Existing research has largely examined health workforce shortages, motivation, retention, or specific cadres in isolation. Few studies have adopted a systems perspective to examine how education, competencies, workplace exposure, labour market conditions, and institutional factors collectively influence youth employment readiness within the PHC ecosystem. Furthermore, evidence from Zambia integrating the perspectives of trainees, educators, employers, regulators, and early-career professionals remains limited. Addressing this evidence gap is essential for informing policies that simultaneously strengthen the health workforce and improve employment opportunities for young people.

Therefore, this study aimed to identify the institutional and individual-level barriers and facilitators influencing youth employment readiness within Zambia's primary health care ecosystem. Specifically, the study sought to: (i) assess employment readiness among PHC trainees and early-career professionals; (ii) explore barriers to recruitment, workforce entry, and retention; (iii) identify opportunities for strengthening youth employment pathways within Zambia's PHC workforce, and (iv) identify factors associated with employment readiness.

## 2 Materials and methods

This study is a secondary analysis of quantitative and qualitative data collected as part of the Africa Health Collaborative, a comprehensive mixed-methods cross-sectional study conducted to identify barriers and facilitators to health employment, health entrepreneurship, and health ecosystem strengthening. The broader needs assessment employed a convergent mixed-methods design in which quantitative surveys with 859 respondents i.e., 770 students and 89 PHC workers, and qualitative methods involving 64 key informant interviews (KIIs), and 5 focus group discussions (FGDs) were conducted concurrently and integrated during interpretation through triangulation. Although the needs assessment addressed three programme pillars (Health Employment, Health Entrepreneurship and Health Ecosystem), this manuscript reports findings from the Health Employment (HEMP) pillar.

The study was conducted in four districts in Zambia (Lusaka, Kafue, Kitwe and Ndola), selected because they host major public and private health professional training institutions and health facilities involved in the education, internship and employment of primary healthcare workers. Data were collected from nine tertiary training institutions and four health facilities offering internship and early-career placement opportunities for PHC professionals. Targeted programmes included medicine, nursing, public health, environmental health, pharmacy, clinical medicine, physiotherapy, midwifery, and other PHC-related disciplines.

The quantitative component included final-year students enrolled in PHC-related training programmes, interns, volunteers, and early-career health professionals. The qualitative component included academic leaders, heads of schools and departments, employers, human resource managers, regulatory authorities and professional associations involved in health workforce development.

The quantitative component of this study utilized data from the Health Employment (HEMP) pillar of the Africa Health Collaborative. The sample size for the broader needs assessment was determined during programme design to ensure adequate representation of the targeted respondent groups across the participating institutions and study districts, and to support subgroup analyses and multivariable statistical modelling. Guided by the Central Limit Theorem, a minimum of 30 respondents was targeted within each sampling unit to support reliable statistical inference, while the overall sample was proportionately allocated according to programme enrolment across participating institutions.

A multistage sampling approach was employed. First, tertiary training institutions and participating health facilities were purposively selected based on their involvement in training and employing primary health care professionals within the four study districts. Subsequently, eligible participants were selected using systematic random sampling from student enrolment registers, internship registers, and staff lists. The sampling interval for each institution or facility was determined by dividing the total number of eligible individuals by the allocated sample size for that institution, after which every *k*th eligible participant was selected. Where a selected individual declined participation or was unavailable, the next eligible individual on the sampling frame was recruited.

The present study analysed data from 859 respondents, representing participants with complete information on the outcome variable and explanatory variables included in the multivariable logistic regression analysis. Records with incomplete or missing data on variables required for the regression model were excluded from the analytical dataset.

Quantitative data was collected using a structured questionnaire programmed in KoboToolbox. The questionnaire was developed as part of the Africa Health Collaborative based on the study objectives, conceptual framework and existing literature on health workforce development, competency gaps and employment pathways. It captured information on socio-demographic characteristics, educational background, employability, leadership competencies, technical competencies, workplace readiness, career aspirations and perceived barriers and facilitators to employment. Draft questionnaires were reviewed by subject-matter experts from Amref Health Africa and Amref International University to establish content validity before electronic deployment. Qualitative data were collected through 64 key informant interviews and 5 focus group discussions using semi-structured interview guides.

The primary outcome variable was employment readiness. Employment readiness was operationalised as a composite binary indicator intended to capture both labour market preparedness and basic job-seeking competency. Respondents who reported that they felt prepared for the labour market and had developed a curriculum vitae (CV) were classified as employment ready, whereas respondents who did not meet both criteria were classified as not employment ready.

Quantitative data were extracted from KoboToolbox and imported into Stata version 17 for analysis. Descriptive statistics were used to summarise respondent characteristics. Associations between employment readiness and explanatory variables were first examined using Pearson's Chi-square tests. Variables associated with the outcome at *p* < 0.20, together with variables identified *a priori* from the conceptual framework and previous literature, were considered for inclusion in the multivariable logistic regression model. A backward elimination approach was used to obtain the final model while retaining variables considered conceptually important. Multicollinearity among predictor variables was assessed using variance inflation factors (VIFs). Model fit was evaluated using the Hosmer–Lemeshow goodness-of-fit test and the likelihood ratio test. Associations were reported as adjusted odds ratios (AORs) with 95% confidence intervals, and statistical significance was determined at *p* < 0.05. Quantitative findings were subsequently interpreted alongside qualitative findings to identify areas of convergence and complementarity through triangulation.

Respondents rated their confidence across six technical competency domains using a four-point Likert scale (1 = Not at all confident, 2 = Slightly confident, 3 = Confident, and 4 = Highly confident). Internal consistency of the competency scale was assessed using Cronbach's alpha, with values ≥0.70 considered acceptable. Mean scores were calculated for each competency domain. An overall mean competency score (grand mean) was then computed as the arithmetic mean of the six domain-specific mean scores to provide an overall summary of respondents' perceived technical competence. Competency domains with mean scores above the overall mean were interpreted as relatively stronger competencies, whereas those below the overall mean indicated priority areas for strengthening.

Key informants and focus group participants were purposively selected based on their roles in health workforce education, regulation, and employment. Qualitative interviews and focus group discussions were audio-recorded with participant consent, transcribed verbatim, and analysed using ATLAS.ti. An inductive-deductive thematic analysis approach was employed. Initial codes were developed from the study objectives and conceptual framework and refined iteratively as new themes emerged from the data. The qualitative findings were integrated with quantitative results during interpretation to explain statistical associations and provide a more comprehensive understanding of barriers and facilitators to youth employment in primary healthcare.

Ethical approval for the Africa Health Collaborative was obtained from the National Health Research Authority of Zambia (Reference No. NHRA-1562/17/09/2024). Additional approvals were obtained from participating training institutions and provincial health authorities. Written informed consent was obtained from all participants before data collection. All study personnel signed confidentiality agreements, and electronic data were securely stored with access restricted to the research team.

## 3 Results

### 3.1 Socio-demographic characteristics and youth employment of the respondents

The study included a total of 859 respondents for the quantitative survey and 64 key informant interviews and 5 focus group discussions for the qualitative methods, with representation across four districts. The quantitative sample was predominantly drawn from Lusaka (51.8%), followed by Kafue (21.3%), Kitwe (15.8%), and Ndola (11.1%), indicating a strong urban concentration. In terms of age distribution, the respondents were largely young, with a mean age of 25.2 years (SD: 3.8). More than half (51.9%) were aged 19–24 years, followed by 37.5% aged 25–29 years, while only a small proportion were aged 30 years and above (10.6%), confirming a predominantly youth sample. Regarding sex distribution, the sample was female-majority (58.2%), compared to 41.8% male participants. In terms of education level, over half of the respondents (55.1%) had diploma-level or lower qualifications, while 44.9% had undergraduate education or higher, suggesting a relatively well-educated cohort. Looking at health cadre/career status, the majority of participants were students (84.5%), with smaller proportions being early-career professionals or volunteers (10.4%) and interns (5.1%), indicating that the sample is largely composed of individuals still in training. With respect to mode of learning, most respondents reported in-person training (55.7%), followed by blended learning (44.0%), while purely online learning was negligible (0.4%).

Table 1 summarizes the distribution of youth employment readiness across demographic, educational, and contextual characteristics.

**Table 1:** Sociodemographic characteristics of the respondents by youth employment (*N* = 859).

Variable	Youth Employment		Total *n* (%)	*p*-value
	Not Employment-Ready *n* (%)	Employment Ready *n* (%)		
Sex				
Female	299 (60)	201 (40)	500 (100)	0.34
Male	203 (57)	156 (43)	359 (100)	
Marital Status				
Ever married	23 (32)	47 (67)	70 (100)	<0.001
Single	479 (61)	310 (39)	789 (100)	
Age				
18–24	326 (73)	120 (27)	446 (100)	<0.001
25–29	150 (47)	172 (53)	322 (100)	
30–34	15 (25)	44 (75)	59 (100)	
35+	11 (34)	21 (66)	32 (100)	
Geographic setting				
Rural	57 (63)	33 (37)	90 (100)	0.28
Peri-urban	84 (63)	50 (37)	134 (100)	
Urban	361 (57)	274 (43)	635 (100)	
Level of Education				
Diploma	333 (70)	140 (30)	473 (100)	<0.001
Undergraduate or Over	169 (44)	217 (56)	386 (100)	
Career Status				
Early career/Volunteer	11 (12)	78 (88)	89 (100)	<0.001
Finalist student	481 (66)	245 (34)	726 (100)	
Intern	10 (23)	34 (77)	44 (100)	
Mode of Learning				
Blended	184 (49)	194 (51)	378 (100)	<0.001
In-person	317 (66)	161 (34)	478 (100)	
Purely Online	1 (33)	2 (67)	3 (100)	
District				
Kitwe	100 (74)	36 (26)	136 (100)	<0.001
Ndola	40 (42)	55 (58)	95 (100)	
Lusaka	247 (56)	198 (44)	445 (100)	
Kafue	115 (63)	68 (37)	183 (100)	
Total	502 (58)	357 (42)	859 (100)	

Values are presented as *n* (%). Percentages are calculated within each category. Employment readiness was operationalised as a composite binary outcome based on respondents who reported both feeling prepared for the labour market and having developed a curriculum vitae (CV). *P*-values were obtained using Pearson's Chi-square tests.

There were no significant sex differences in employment readiness, with 40% of females and 43% of males classified as employment-ready (*p* = 0.34). In contrast, marital status was strongly associated with employment readiness (*p* < 0.001): 67% of ever-married youth were employment-ready compared to only 39% of single youth. Age was also a significant predictor (*p* < 0.001), with readiness increasing from 27% among those aged 18–24 years to 53% in the 25–29 age group, 75% among 30–34-year-olds, and 66% in those aged 35 years and above. Geographic setting showed no significant association with employment readiness (*p* = 0.28), with comparable proportions in rural (37%), peri-urban (37%), and urban (43%) areas. Educational attainment was significantly related to employment readiness (*p* < 0.001). More than half (56%) of youth with an undergraduate degree or higher were work-ready, compared to 30% among those with a diploma. Employment readiness differed significantly by engagement in work-based roles. Respondents involved in volunteer work (88%) or internships (77%) were far more likely to be employment-ready than finalist students (34%, *p* < 0.001). Mode of learning was also significantly associated with employment readiness (*p* < 0.001). Youth participating in blended learning (51%) or online-only programs (67%) were more likely to be employment-ready than those in fully in-person learning (34%). Additionally, employment readiness varied significantly across districts (*p* < 0.001), indicating potential local or institutional differences in opportunities for preparing youth for the workforce. The highest proportion of employment-ready youth was observed in Ndola (58%), followed by Lusaka (44%) and Kafue (37%), while Kitwe had the lowest proportion (26%). Specifically, older youth, those with higher education, and those who were already in volunteer/early career roles reported higher levels of employment readiness compared to younger, less educated, and student respondents.

### 3.2 Employment preparedness

Among the students, volunteers, and interns who reported being prepared for the job market from the focus group discussions, various reasons were provided for this preparedness. Some reported being prepared for employment due to the knowledge gained during their training. They mentioned understanding both theory and its clinical application including diagnosis, treatment, patient care, and disease prevention. Their courses were seen as largely relevant and sufficient for professional roles. Hands-on experience during clinical attachments, internships, and volunteer work greatly contributed to their confidence. Some said they had the opportunity to perform key procedures and work closely with patients, which helped bridge the gap between learning and practice. The respondents felt that possession of soft skills such as communication, leadership, and adaptability was a sort of preparedness for the job market. Mentorship from instructors and supervisors played a key role in building competence. Constructive feedback during attachments helped improve their practical skills and professionalism. They expressed personal confidence, a passion for their work, and a desire to serve communities as a facilitator to job preparedness. Some cited previous work or diploma experience as added preparation as well as community health readiness, including public health education and outreach.

### 3.3 Pre-service training (knowledge and competencies and skills)

A total of 468 (54.5%) respondents felt very knowledgeable; in their general areas of training in PHC 300 (34.9%) were moderately knowledgeable, while 76 (8.9%) were extremely knowledgeable. Only 15 (1.8%) reported to be slightly knowledgeable. (Table 2).

**Table 2:** Self-reported training knowledge levels by cadre, career status and mode of learning (*N* = 859).

Category	Slightly knowledgeable *n* (%)	Moderate knowledgeable *n* (%)	Very knowledgeable *n* (%)	Extremely knowledgeable *n* (%)
Cadre				
Doctors, CO's, pharmacists and dentists	4 (1.4)	122 (35.4)	190 (55.1)	28 (8.1)
Laboratory services	0	9 (24.3)	24 (64.9)	4 (10.8)
Nurses	4 (1.5)	111 (40.5)	131 (47.8)	28 (10.2)
Nutritionists, public health & community health	6 (3.6)	41 (24.7)	106 (63.9)	13 (7.8)
Technical	0	17 (45.9)	17 (45.9)	3 (8.1)
Learning status				
Early career	1 (1.2)	25 (28.1)	49 (55.1)	14 (15.7)
Finalist	14 (1.9)	263 (36.2)	392 (54)	57 (7.8)
Intern	0	12 (27.3)	27 (61.4)	5 (11.4)
Mode of learning				
Blended learning (in person and online)	6 (0.7%)	123 (14.3%)	218 (25%)	31 (3.6%)
Online	0	1 (0.1%)	1 (0.1%)	1 (0.1%)
In-person	9 (1.0%)	174 (20.3%)	243 (28.3%)	41 (4.7%)
Interns and early career	0	2 (0.2)	6 (1%)	3 (0.3%)
General areas of training	15 (1.8)	300 (34.9)	468 (54.5)	76 (8.9)

Respondents rated their perceived level of training knowledge using a four-point Likert scale ranging from 1 = Slightly knowledgeable to 4 = Extremely knowledgeable. Values are presented as *n* (%). Percentages for cadre and career status are calculated within each subgroup, while percentages for mode of learning are based on the overall study sample.

Laboratory services (64.9%), Nutritionists, public health & community health (63.9%), and Doctors, CO's, pharmacists and dentists (55.1%) were the cadres who led the pack in exhibiting “very knowledgeable” in their general area of training.

Female respondents were very knowledgeable (56.2%) compared to male (43.8%). At the same time, female respondents were extremely knowledgeable (61.8%) compared their male Counterparts (38.2%).

The respondents were asked to evaluate themselves on competencies. The six-item technical competency scale demonstrated acceptable internal consistency (Cronbach's *α* = 0.78), indicating good reliability of the items used to measure perceived technical competence. Respondents rated their confidence on a four-point Likert scale ranging from 1 (Not at all confident) to 4 (Highly confident). The overall mean competency score across the six domains was 2.21, suggesting that, on average, respondents perceived themselves as being slightly confident and confident in their technical competencies. Competency domains with mean scores above 2.21 were interpreted as relative strengths, whereas those scoring below the overall mean were considered areas requiring additional training and support. The respondents exhibited high confidence in their communication and critical thinking skills (Table 3). However, their confidence was notably lower in areas such as leadership, conflict resolution, technical skills, and networking.

**Table 3:** Level of confidence of the respondents in different competencies by gender.

Competency Area	Mean	Interpretation	Mean	Interpretation
	Female		Male	
Technical skills	2.15	Low confidence	2.13	Low confidence
Leadership skills	2.19	Low confidence	2.21	High confidence
Communication skills	2.42	High confidence	2.42	High confidence
Critical thinking skills	2.26	High confidence	2.24	High confidence
Conflict resolution and interpersonal skills	2.18	Low confidence	2.21	High confidence
Networking skills	2.08	Low confidence	2.05	Low confidence

Respondents rated each competency using a four-point Likert scale (1 = Not at all confident, 2 = Slightly confident, 3 = Confident, and 4 = Highly confident). Cronbach's alpha for the six-item scale was 0.78, indicating acceptable internal consistency. The overall mean competency score (grand mean) across all six competency domains was 2.21.

There are gender differences in confidence levels across competencies. Both genders showed high confidence in communication (2.42) and critical thinking skills (female 2.26, male 2.24). Males had higher confidence in leadership (2.21) and conflict resolution (2.21) compared to females (2.19 and 2.18) Table 4. Both genders had low confidence in technical skills (female 2.15, male 2.13) and networking skills (female 2.08, male 2.05), with females slightly higher. The overall average confidence scores for both genders were equal at 2.21. Addressing these disparities in training is crucial for balanced competency development.

**Table 4:** Logistic regression predicting youth employment readiness (*N* = 859).

Variable	Odds Ratio (OR)	95% Confidence Interval	*p*-value	Significance
Age Category (ref: 19–24)				
25–29	2.05	[1.45, 2.89]	<0.001	***
30–34	4.31	[2.13, 8.70]	<0.001	***
35+	3.28	[1.43, 7.54]	0.005	**
Career Status (ref: Student)				
Volunteer/Early career	17.55	[8.56, 36.0]	<0.001	***
Intern	2.97	[1.39, 6.38]	0.005	**
Education Level (ref: Diploma)				
Undergraduate or higher	2.91	[1.89, 4.48]	<0.001	***
District (Kitwe)				
Kafue	3.22	[1.69, 6.12]	<0.001	***
Lusaka	2.49	[1.32, 4.69]	<0.001	***
Ndola	2.11	[0.97, 4.62]	0.060	
Constant	0.09	[0.05, 0.16]	<0.001	***
Model Statistics	Significance levels			
Log likelihood: –465.35	* *p* < 0.05			
LR chi^2^ (9): 235.52	** *p* < 0.01			
*p*-value: <0.000	*** *p* < 0.001			
Pseudo R^2^: 0.2019				
Hosmer–Lemeshow chi^2^ (6) = 2.01				
Prob > chi^2^ = 0.9191				

### 3.4 Barriers to market readiness

The reasons given for lack of preparedness by students, interns, volunteers, and early careers included lack of practical experience especially uncertain about performing certain procedures or handling real patients. Others pointed to the short duration or limited quality of attachments, saying they didn't cover enough ground. A few mentioned that the training environment differed greatly from real-world settings, which created anxiety about transitioning into the workforce. Some respondents expressed psychological unpreparedness, citing fear, anxiety, and lack of confidence. Others felt that the curriculum was outdated, too rushed, or didn't fully align with industry demands.

“With the local training, the practical part is not that much, it lacks such that when you go for attachment or even internships the people that you find there will really tell you to say what you need is the practical not the theory that you learnt in class. So, if even them they know that what we learn in class and what we practice is actually different. I feel like we should in cooperate more of the practice in our training because it the practical work that we really need not the theory.” FGD Participant, Final year Students, Alumni, Interns and Early Career.

A few were still in training or had not yet completed final rotations or internships and so were not adequately prepared for the job market.

From key informant interviews, training quality has declined due to commercialization, with a focus on quantity over standards. Poor regulation, inadequate infrastructure, and a surge in nursing colleges have led to underprepared graduates. Many students prioritize completing school over gaining practical skills, relying on dummy-based training instead of real clinical experience.

With the mushrooming colleges of nursing that we have currently, the ones that graduate and are picked to come and work even as volunteers, they are not giving us that technical know-how. The skill is not there. For most of them, I shouldn't just say for some of them, for a lot of them—Employer, Zambia, KII

But then I think some of these tertiary institutions are just doing it for money. The feasibility of those programs is not enacted actually, so half-baked students or literally, there not even baked at all … Employer Zambia, KII

### 3.5 Opportunities for youth in PHC

Young people identified their preferred priority workplaces in PHC as Hospital facility (80.7%), NGOs (66.5%), Community settings (40.5%) and working overseas (30.5%). Conversely, Supply chain (7.1%), health insurance (8.1%) and education institutions (26.5%) where least areas respondents would wish to apply their professional skills (Figure 1: Preferred work places for young PHC workers).

**Figure 1:**
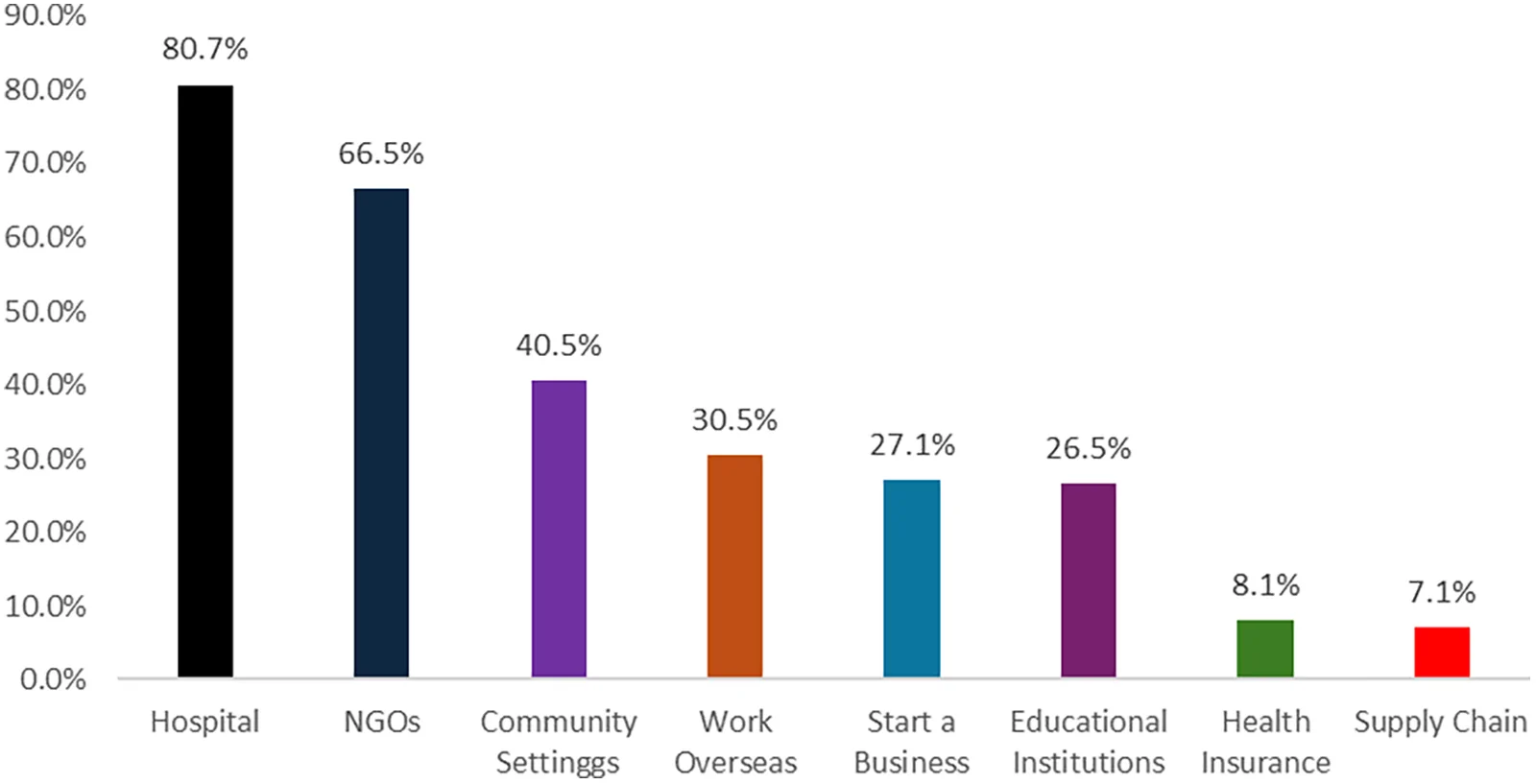
Preferred work places for young PHC workers.

The young people identified job opportunities for the non-health sector professionals can work in support the health sector such as health education and community awareness where teachers, media personnel, and community leaders can engage in public sensitization and awareness campaigns. These professionals, though not trained in health, can play important roles in disseminating accurate health information, promoting healthy behaviors, and creating supportive environments for health service uptake.

Beyond the typical health sector, respondents expressed a wide range of preferred work areas beyond clinical practice. There is an interest to work in the mining and industrial sectors, particularly in roles related to occupational health, safety, and environmental management.

Some respondents preferred to be self-employed, aiming to run businesses, farms, or private clinics. A few expressed interests in sustainable development, public speaking, or policy-level work that influences systems and structures. The young people expressed interest to work in sports and fitness therapy, massage parlors, military camps, prisons, restaurants, and social welfare institutions were mentioned.

### 3.6 Employment and retention barriers of PHC workers

Qualitative findings indicated that participants viewed recruitment, workforce entry and retention as interconnected components of the youth employment pathways. Key informant interviews with academic leaders, regulators, and employers identified barriers to youth employment and retention in PHC services as poor working conditions, inadequate infrastructure, lack of essential resources, and overwhelming workloads due to staff shortages. These barriers lead to burnout and dissatisfaction among health workers. Geographical location also plays a role because many facilities in remote or underserved areas often have dilapidated infrastructure thus discouraging long-term commitment.

“The standard issue is the conditions of service, growth opportunities, hence, the brain drain that takes place, but also the capacity of the public services.”—Employer Zambia, KII

“In terms of the location, geographical location. Most of these primary health care facilities, the working conditions, you know, they you would find some. Some of these health facilities are dilapidated houses and so on. So just I would really look at geographical location as well as the working conditions, infrastructure and so on.”—Employer Zambia, KII

These barriers are represented in a word cloud on Figure 2.

**Figure 2:**
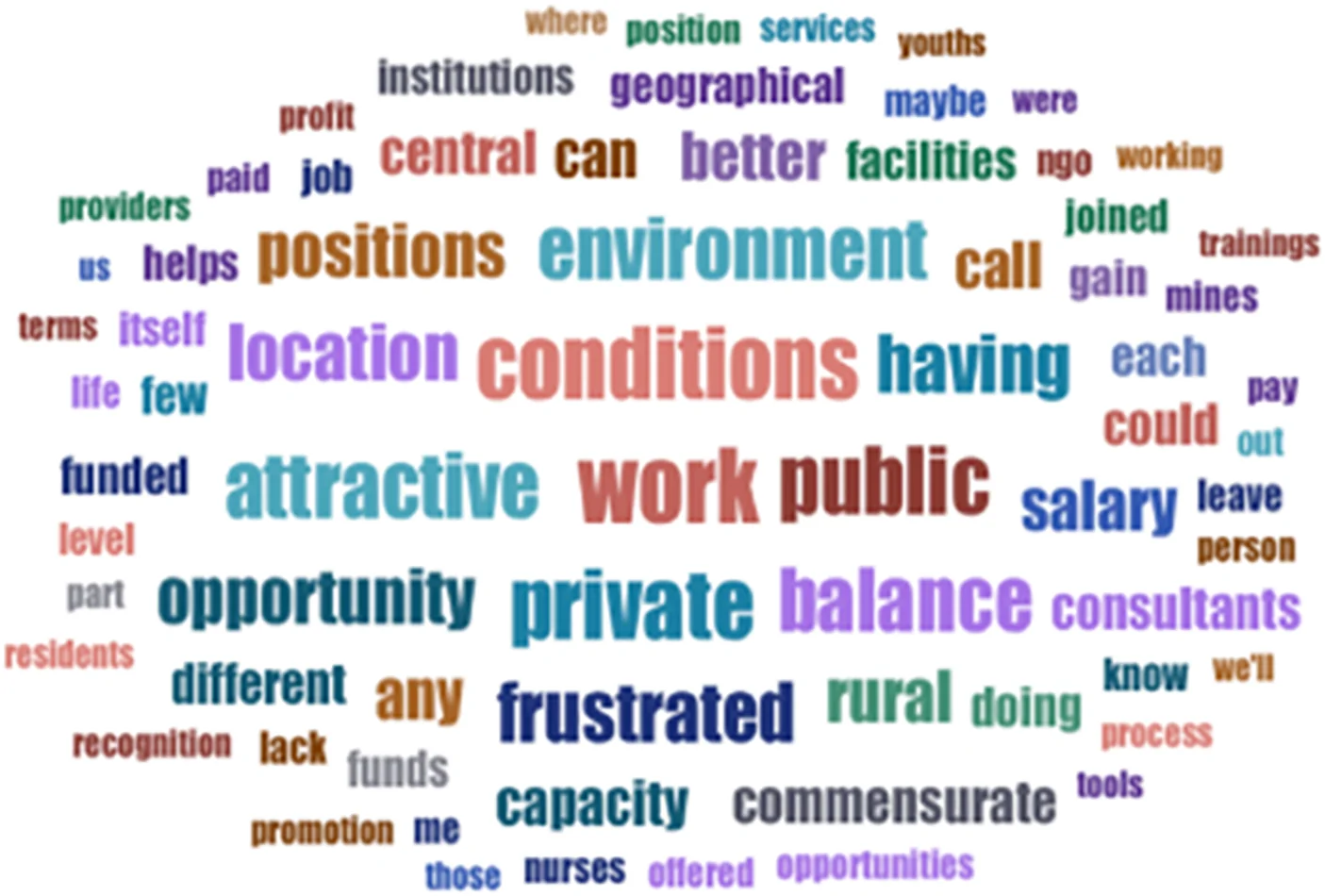
Word cloud of employment and retention barriers of PHC workers.

Low remuneration and lack of financial incentives such as housing or hardship allowances significantly impact retention. Employees often feel their compensation does not match their workload or cost of living, making it difficult to sustain their roles, especially in rural settings. Some contractual instabilities such as delays in absorbing volunteers or lack of funds to maintain contracts were also included by the key informants. A lack of career progression opportunities and limited support for further education also undermine retention. Many workers face barriers such as absence of study leave, lack of sponsorship, and minimal institutional support for upgrading qualifications. This contributes to a feeling of stagnation and prompts health professionals to leave for better prospects. Work-life balance challenges further affect retention. Without supportive policies such as adequate leave, flexible scheduling, and recognition of family dynamics, employees struggle to maintain both personal and professional stability. Comparatively, NGOs tend to retain staff better due to more attractive conditions, including education opportunities and structured work-life support. Lack of staff recognition, motivation, and mentorship reduces morale. When employees feel undervalued or unsupported in their growth and wellbeing, it weakens their attachment to their roles, increasing turnover.

“we've seen a lot of human resource for health attrition and this is primarily attributed to the fact that most of our people or healthcare providers feel their pay is not commensurate, that's one component. The salaries or the emoluments are not commensurate to the work they put in”—Health Association Leader, KII

### 3.7 Youth employment readiness and social demographic characteristics

To identify independent predictors of youth employment readiness, a multivariate logistic regression model was fitted (Table 2). After adjustment for covariates, age remained a strong predictor of employment readiness: participants aged 25–29 (OR = 2.05, *p* < 0.001), 30–34 (OR = 4.31, *p* < 0.001), and 35+ (OR = 3.28, *p* = 0.005) all showed significantly higher odds of employment readiness compared to those aged 19–24. Career status also remained highly predictive, with volunteers/early career respondents reporting more than 17-fold higher odds (OR = 17.55, *p* < 0.001), and interns nearly threefold higher odds (OR = 2.97, *p* = 0.005), relative to students. Education maintained a strong association, with undergraduates and above being nearly three times more likely to be employment-ready (OR = 2.91, *p* < 0.001) than diploma holders.

District effects were partly sustained: youth from Kafue (OR = 3.22, *p* < 0.001) and Lusaka (OR = 2.49, *p* = 0.005) had higher odds of employment readiness relative to the reference district (Kitwe), while the effect for Ndola, though elevated (OR = 2.11), did not remain statistically significant (*p* = 0.060).

The final model showed good explanatory power [LR *χ*^2^(9) = 235.52, *p* < 0.001, Pseudo R^2^ = 0.2019] and was well calibrated according to the Hosmer–Lemeshow goodness-of-fit test [*χ*^2^(6) = 2.01, *p* = 0.919].

## 4 Discussion

The findings of this study demonstrate a fair level of employment readiness among youth in PHC in Zambia. The findings indicate that youth employment readiness within Zambia's PHC ecosystem was more strongly associated with life stage, educational progression, and institutional exposure than with gender. While no significant differences were observed by sex or rural–urban setting, employment readiness increased markedly with age, higher education levels, and participation in volunteer or early-career roles. These patterns suggest that structural pathways into the workforce, rather than individual demographic characteristics alone, may play an important role in shaping employment readiness.

Consistent with prior research (10, 11), these findings indicate that youth employability and transition into the health workforce are associated with life stage, educational attainment, and workplace exposure rather than sex or geographic location. It is important to note, however, that employment readiness in this study reflects participants' self-reported preparedness for entering the labour market rather than objectively measured competence or subsequent employment outcomes. Consequently, the observed associations should be interpreted as reflecting perceived readiness rather than verified employability.

Educational attainment was significantly associated with employment readiness, with diploma holders reporting lower employment readiness than those with undergraduate or higher qualifications. Qualitative insights suggest that this association may reflect, in part, differences in curriculum depth, exposure to analytical and leadership skills, and the quality of clinical attachments. The rapid expansion and commercialization of health training institutions in Zambia and the wider region have outpaced regulatory capacity, resulting in variable training quality and the graduation of underprepared students (10, 14). These challenges may contribute to lower employment readiness among diploma holders and other graduates, as they may have had less exposure to critical clinical, analytical, and leadership competencies required in the workplace. Gendered differences observed in self-reported confidence (perceived competence) across leadership, conflict resolution, and technical competencies are consistent with literature highlighting structural and institutional factors shaping leadership opportunities, which in turn influence self-perceived competence within health systems (13).

Together, these findings underscore the need for competency-based training programmes and gender-responsive mentorship models that address observed gaps in confidence and leadership skills, ensuring that all graduates, regardless of gender, are adequately prepared to meet workforce demands. More robust accreditation and enforcement of training standards may further strengthen alignment between training institutions and employer requirements, thereby improving graduates' employment prospects.

One of the most striking findings was the strong association between employment readiness and engagement in internships, volunteering, or early-career roles. Youth in these categories were significantly more likely to report employment readiness than students still in training. This aligns with international evidence that structured school-to-work transition mechanisms, such as internships, supervised practice, and mentorship, are associated with the development of confidence, practical skills, and professional identity among health workers (15).

In the Zambian context, studies have identified delays in internship placement, limited absorption capacity, and inconsistent funding for volunteer positions as important bottlenecks to health workforce entry, particularly in public-sector PHC settings (12). The present findings suggest that expanding and formalizing these transition pathways could strengthen workforce entry and should be considered as part of broader workforce development strategies rather than *ad hoc* arrangements.

The association between blended or online learning and higher self-reported employment readiness suggests that exposure to flexible and technology-enabled learning may enhance adaptability, self-directed learning, and digital competencies—skills that are increasingly valued in modern health systems. This finding aligns with broader literature on early-career professional development and communities of practice, which emphasizes adaptability, peer learning, and reflective practice as key employability enablers in health systems (15).

However, district-level differences in learning modes point to persistent infrastructure and connectivity gaps, particularly outside major urban centres. Without deliberate investment in digital infrastructure and faculty capacity, expanded online learning may risk reinforcing existing inequities rather than reducing them.

While entering the workforce is an important milestone, retention of health workers in PHC services is constrained by poor working conditions, inadequate infrastructure, low remuneration, excessive workloads, and limited opportunities for career advancement. Participants consistently identified peer pressure and the pursuit of higher compensation in private or NGO settings as factors influencing decisions to leave public-sector employment, whereas recognition, supportive supervision, and professional development opportunities were viewed as important incentives for retention. Nevertheless, systemic challenges, including low pay and inadequate infrastructure, were perceived to outweigh these positive drivers, limiting overall workforce stability (16). These factors have been widely associated with dissatisfaction, attrition, and internal or external migration among health workers in LMICs (11, 17). Comparatively better retention in NGO settings is consistent with broader evidence that supportive supervision, access to continuing education, and work–life balance policies are associated with workforce retention in PHC (12). Addressing these issues within the public sector may help reduce internal migration and external brain drain, which continue to undermine health system resilience (17, 18).

By 2025, Zambia is projected to face a substantial health workforce gap. Based on a combined health workforce ratio of 4.1 per 1,000 population, the country will require approximately 91,554 health workers. Considering the current stock of 44,583, anticipated inflows of 17,976, and projected attrition of 8,649, the estimated shortfall is 37,644 health workers (7). This projected deficit underscores the urgency of strengthening both recruitment and retention strategies, particularly in primary health care settings, to support the country's capacity to meet population health needs and sustain service delivery.

Importantly, the study highlights that PHC employment opportunities extend beyond traditional clinical roles. Youth expressed interest in health-adjacent and non-health occupations such as health education, occupational health, community outreach, entrepreneurship, and policy-oriented roles. This aligns with global evidence demonstrating that for every health occupation (HO) worker, multiple non-health occupation (NHO) roles are generated within the health ecosystem, particularly in low-income settings (3, 19, 21).

Recognizing PHC as an employment ecosystem rather than a narrow clinical sector has important policy implications. Strengthening career guidance, entrepreneurship training, and linkages with non-traditional employers could help absorb a growing youth population while supporting broader health system goals (20).

Taken together, the findings suggest that improving youth employment outcomes in PHC requires a systems-level response. Investments should move beyond increasing training output to focus on quality, relevance, and absorption capacity (22). Priority actions include strengthening regulation of training institutions, expanding structured internship and mentorship pathways, integrating leadership and soft skills into curricula, improving working conditions, and deliberately promoting health-adjacent employment opportunities. Such reforms are consistent with the WHO's Global Strategy on Human Resources for Health: Workforce 2030, which emphasizes alignment between education, labour markets, and service delivery needs (2).

## 5 Strengths and limitations

A key strength of this study lies in its inclusive and mixed-methods design, which brought together perspectives from across the full PHC employment pipeline. The study engaged a wide range of stakeholders directly involved in the education, regulation, employment, and supervision of health workers, including heads of training institutions and academic departments, lecturers, human resource managers, regulatory authorities, student leaders, and PHC worker unions. In addition, the study incorporated voices of future, transitioning, and early-career PHC workers, ranging from students and recent graduates to interns, volunteers, and early-career professionals.

This breadth of perspectives enabled a comprehensive examination of the transition from education to practice, capturing both supply-side (training quality, curriculum relevance, skills acquisition) and demand-side (employer expectations, working conditions, retention incentives) dimensions of youth employment in PHC. The integration of quantitative survey data with rich qualitative insights strengthened the validity of the findings through triangulation, allowing observed statistical associations, such as the role of age, education, and early-career exposure in employment readiness, to be contextualized within lived experiences and institutional realities.

Furthermore, the inclusion of multiple districts with varying urban and peri-urban characteristics enhanced the contextual relevance of the findings and their applicability to diverse PHC settings in Zambia. By grounding the analysis in experiences across different stages of professional development and institutional roles, the study provides policy-relevant evidence that is both empirically robust and practically grounded.

Some of the limitations that should be considered when interpreting the findings include the following; First, the cross-sectional design captures perceptions of employment readiness, skills, and barriers at a single point in time, limiting the ability to infer causal relationships or assess changes in readiness and employment outcomes over time. Longitudinal studies following cohorts of students and early-career professionals from training through employment would provide stronger evidence on pathways into the PHC workforce and determinants of sustained employment.

A further limitation relates to the measurement of employment readiness, which was based on self-reported perceptions rather than objective assessments of employability or subsequent employment outcomes. Self-reported measures are susceptible to social desirability bias, whereby respondents may overstate their preparedness to align with perceived expectations, as well as response bias, where confidence may not accurately reflect actual competence or job readiness. Consequently, the employment readiness measure should be interpreted as reflecting perceived readiness rather than objectively verified employability. Although triangulation with qualitative findings and employer perspectives strengthened the interpretation of these results, future research should incorporate objective indicators such as competency assessments, employment outcomes, employer evaluations, or longitudinal follow-up to validate self-reported readiness over time.

Third, while the study included multiple districts and a diverse range of stakeholders, the selected districts were purposively chosen based on programmatic relevance and may not fully represent all PHC contexts in Zambia, particularly remote rural settings. As such, the generalizability of findings to districts with different health system capacities or labour market dynamics may be limited.

## 6 Conclusion

This study provides robust evidence on the barriers and opportunities shaping youth employment readiness within Zambia's primary health care ecosystem. While Zambia faces high overall youth unemployment, the PHC sector remains a viable and underutilized avenue for generating meaningful employment for both health and non-health occupations. Employment readiness among youth is strongly influenced by age, education level, and exposure to real-world work environments, rather than by sex or geographic location alone.

The findings point to persistent gaps in pre-service training quality, clinical exposure, leadership development, and market-aligned skills, compounded by weak regulation and limited transition-to-work opportunities. At the same time, poor working conditions, low remuneration, and constrained career progression undermine retention within the PHC workforce. Addressing these challenges requires coordinated action across training institutions, regulatory bodies, employers, and government.

Policy and programmatic responses should prioritize strengthening accreditation and quality assurance of training institutions; expanding structured internships, mentorships, and volunteer-to-employment pathways; integrating leadership, entrepreneurship, and soft skills into curricula; and improving working conditions and incentives, particularly in underserved areas. Equally important is broadening career guidance to reflect the full spectrum of opportunities within the PHC ecosystem, including health-adjacent and entrepreneurial pathways.

By aligning youth development strategies with health workforce needs, Zambia can harness its youthful population to build a resilient, responsive PHC system while simultaneously advancing national employment and development goals. The evidence generated by this study provides a strong foundation for informing workforce reforms, targeted investments, and future research aimed at strengthening the health employment ecosystem in Zambia.

## Data Availability

The raw data supporting the conclusions of this article will be made available by the authors, without undue reservation.
